# The mental health and well-being of internally displaced female Yazidis in the Kurdistan Region of Iraq: a realist review of psychosocial interventions and the impact of COVID-19

**DOI:** 10.1017/gmh.2022.55

**Published:** 2022-11-04

**Authors:** Sophia Lobanov-Rostovsky, Ligia Kiss

**Affiliations:** Institute for Global Health, University College London, London, WC1N 1EH, UK

**Keywords:** gender-based violence, internally displaced person, psychosocial, post-traumatic stress disorder, Yazidi

## Abstract

**Background:**

Yazidis in the Kurdistan Region of Iraq have been exposed to recurrent traumatic experiences associated with genocide and gender-based violence (GBV). In 2014, ISIS perpetrated another genocide against the Yazidi community of Sinjar. Women and girls were held captive, raped and beaten. Many have been forced into displacement. Rates of post-traumatic stress disorder (PTSD) and suicide are high. Limited research has evaluated interventions delivered to this population.

**Methods:**

This review explores how the global evidence on psychosocial interventions for female survivors of conflict-related sexual violence applies to the context of the female Yazidi population. We used a realist review to explore mechanisms underpinning complex psychosocial interventions delivered to internally displaced, conflict-affected females. Findings were cross-referenced with eight realist, semi-structured interviews with stakeholders who deliver interventions to female Yazidis in the Kurdistan Region of Iraq. Interviews also allowed us to explore the impact of COVID-19 on effectiveness of interventions.

**Results:**

Seven mechanisms underpinned positive mental health outcomes (reduced PTSD, depression, anxiety, suicidal ideation): safe spaces, a strong therapeutic relationship, social connection, mental health literacy, cultural-competency, gender-matching and empowerment. Interviews confirmed relevance and applicability of mechanisms to the displaced female Yazidi population. Interviews also reported increased PTSD, depression, suicide and flashbacks since the start of the COVID-19 pandemic, with significant disruptions to interventions.

**Conclusion:**

COVID-19 is just one of many challenges in the implementation and delivery of interventions. Responding to the mental health needs of female Yazidis exposed to chronic collective violence requires recognition of their sociocultural context and everyday experiences.

## Background

In the past two decades, conflict-related sexual violence (CRSV) against women and girls has received increased attention from the international community. CRSV is defined as ‘rape, sexual slavery, forced prostitution, forced pregnancy, forced abortion, enforced sterilisation, forced marriage and any other form of sexual violence of comparable gravity perpetrated against women, men, girls or boys that is directly or indirectly linked to a conflict’ (Guterres, 2019). As these forms of violence gain visibility, interventions responding to associated trauma have received greater investment (Tol *et al*., 2013; Wood, 2014).

Violence extends to post-conflict settings, where women and girls are at an increased risk, with severe consequences to their mental health (Hossain *et al*., 2021). This includes pathological distress (fear, sadness, anger, shame, sadness, guilt), anxiety disorders [post-traumatic stress disorder (PTSD)], depression, somatic complaints, substance abuse, suicidal ideation and self-harm. Social consequences include stigma and its sequelae – social exclusion and discrimination from their communities (Ventevogel *et al*., 2015).

Global prevalence of mental health disorders in these settings is estimated at 22.1% (Charlson *et al*., 2019). Although there is some evidence on the magnitude and risks of CRSV, up-to-date evidence on the contextual effectiveness, mechanisms of change and implementation of psychosocial interventions is missing. Evidence is particularly scarce as interventions face many implementation challenges, including scarcity of resources, low awareness about mental health disorders and stigma. As such, common therapeutic interventions might not be feasible or applicable in these contexts (Seidi and Jaff, 2019).

While the realist-informed review by Spangaro *et al*. (2015) on CRSV identified key mechanisms to prevent violence, they did not address context-specific mechanisms for the promotion of mental health amongst survivors. The most recent systematic review of psychosocial interventions for survivors of sexual violence included data ranging from over a decade ago (Tol *et al*., 2013). While the geographical scope and methodological quality of evaluations were limited, results on effectiveness of interventions were encouraging.

## Research context

### Trauma and mental health of the Yazidi population

The Yazidis are a Kurdish ethnoreligious group originating from the Kurdistan Region of Iraq (KRI), Syria, Turkey, Azerbaijan and Armenia (Erdener, 2017). Due to their minority status, Yazidis have experienced persistent marginalisation and oppression during the Ottoman Empire, and more recently, amongst Sunni Muslims (Jäger, 2019).

Experiences of violence include 74 genocides or ‘Fermans’ (Omarkhali, 2016), a word for the decrees which legitimised violence against Yazidis during the Ottoman Empire (Six-Hohenbalken, 2019). The most recent occurred in August 2014, when ISIS launched an attack on the Yazidi community of Sinjar in Northern Iraq (Jäger, 2019). ISIS's attack resulted in the death and kidnapping of 9900 Yazidis. Thousands were beheaded or burned alive; many perished from dehydration in an attempt to flee to Mount Sinjar (Cetorelli *et al*., 2017*b*). These atrocities have reactivated collective memories of previous genocides and displaced 400 000 Yazidis across the KRI (Dulz, 2016).

ISIS's attack was gender-specific: men were executed, boys were taken as child soldiers and women and girls were subjected to sexual slavery (Cetorelli *et al*., 2017*b*). One study amongst many reports an 8-year-old girl who had been raped hundreds of times during her 14-month captivity (Mohammadi, 2016). 92.6% of Yazidi women residing in an internally displaced person (IDP) camp experienced an average of 4.87 acts of violence during captivity (Goessmann *et al*., 2020). Mass killings and graves mean many don't know whether their relatives are still alive (Womersley and Arikut-Treece, 2019).

Conditions of displacement – inadequate water and housing, extreme temperatures, lack of access to basic resources and loss of legal documentation – lead to ongoing stress (Millar and Warwick, 2019). For those who wish to return to Sinjar, a continual threat of violence lingers. ISIS attacks have been documented throughout 2019 and 2020 (Global Network on Extremism & Technology, 2020). Improvised explosive devices are littered amongst destroyed infrastructure (UNMAS, 2019).

PTSD is the most widely reported mental health disorder amongst Yazidis, with an estimated prevalence of between 70 and 90% (Kizilhan and Noll-Hussong, 2020; Richa *et al*., 2020). Some suggest Yazidis are suffering from complex PTSD, owing to their prolonged exposure to multiple traumatic experiences (Hoffman *et al*., 2018). Rates of suicide and attempted suicide are high (Jaff, 2018; UNHCR, 2019). This is likely a gross underestimate due to its associated stigma (United Nations, 2017). Self-immolation has been reported in response to feelings of shame associated with sexual violence (Medicins Sans Frontieres, 2019). Online Supplementary Fig. S1 summarises the interactions between multiple traumatic exposures and their effect on Yazidi survivors' mental health.

### COVID-19

SARS-CoV-2 (COVID-19) was declared a global pandemic on 11 March 2020; its first case was diagnosed in Iraq on 22 February 2020 (Hussein *et al*., 2020; Zhu *et al*., 2020).

Emerging evidence shows increased depression, anxiety, substance abuse and domestic violence since the outbreak (Galea *et al*., 2020; Othman, 2020; Röhr *et al*., 2020). Individuals have been faced with challenges to their psychological well-being – lifestyle changes, living conditions, school closures, movement restrictions and fear about spread of infection (Othman, 2020). Consequences are likely exacerbated in fragile contexts, especially amongst persons with pre-existing mental health conditions (IASC, 2020).

At the time of writing, only one study had examined the effect of COVID-19 on the mental health of a small sample of Yazidis in a camp near Dohuk (*n* = 38 women) (Kizilhan and Noll-Hussong, 2020). Between October 2019 and April 2020, female Yazidis experienced an 11%, 10% and 6% increase in PTSD, anxiety and depression, respectively.

### Objectives

Limited literature has examined the effectiveness of interventions delivered to the displaced Yazidi population. We explore how global evidence on psychosocial interventions for female survivors of CRSV applies to the female Yazidi population. We also explore the implications of COVID-19 on the implementation and effectiveness of these interventions.

## Methods

This study used a mixed-methods approach to explore how interventions work to improve the mental health of Yazidi survivors of CRSV, and in which circumstances. We used a realist review framework to evaluate how psychosocial interventions may trigger specific mechanisms that interact with the local context to produce intended and unintended outcomes, formulated as context-mechanism-outcome (CMO) configurations (De Souza, 2013) (Dalkin, 2015).

Following RAMESES reporting for realist reviews (online Supplementary Table S1), we began with an exploratory scoping of academic databases, PubMed, PubMed Central, EMBASE, MEDLINE, PsychInfo, Scopus and Web of Science, using terms and eligibility criteria in Table 1. Databases were complemented by reference-list and grey literature screening. Scoping allowed us to formulate an initial context-intervention-mechanism-outcome (CIMO) configuration (Table 2). A CIMO was chosen instead of the more conventional CMO as this offers a clearer configuration for analysis of the interactions between interventions and mechanisms (Booth *et al*., 2018).

**Table 1.** Search terms and eligibility criteria

Component	Search terms	Eligibility criteria	
		Included	Excluded
Type of intervention	‘MHPSS’ OR ‘mental health’ OR ‘psychosocial’ OR ‘psychological’	Any publication year due to specificity of topic	Pharmacological interventions
Intervention words	‘Intervention’ OR ‘programme’ OR ‘service’ OR ‘healing’ OR ‘rehabilitation’ OR ‘therapy’		
Type of mental health condition	‘Post-traumatic stress disorder’ OR ‘PTSD’ OR ‘trauma’ OR ‘depression’ OR ‘anxiety’ OR ‘fear’ OR ‘distress’	If intermediate and/or primary outcomes were mental health indicators or constructs	Languages other than English
Setting	‘Post-conflict’ OR ‘conflict affected’ OR ‘humanitarian setting’ OR ‘internally displaced’ OR ‘IDP’ OR ‘camp’ OR ‘settlement’	Only populations living in IDP settings All ages can be affected by conflict and are found in IDP settings	
Location	‘Low and middle income countries’ OR ‘LMIC’ OR ‘low-resource setting’	Grey literature: journal articles, theses, websites, reports, books and news articles	

MHPSS interventions were ‘any type of local or outside support that aims to protect or promote psychosocial well-being and/or prevent or treat mental disorders’ (IASC, 2020). Grey literature was included so as to capture humanitarian work not published in academic journals. Utilising a diverse range of literature also provides an opportunity for richer theory development (Bunn *et al*., 2016). Grey literature sources included: UNHCR, UNFPA, UNICEF, World Health Organization, International Rescue Committee, International Organization for Migration, Médecins Sans Frontières and ALNAP. Due to limitations of the search tools on the ALNAP website, we searched the term ‘Yazidi’, as this was assumed to include all relevant data to the target population.

**Table 2.** Our CIMO configuration

Component	Initial programme theory	Refined programme theory
Context	Conflict-affected females who are internally displaced in low-resource settings. Have experience of historic and collective trauma, social and gendered inequality, live in poor displacement conditions with poor access to services	
Intervention	Group and individual psychotherapy (narrative exposure therapy, cognitive behavioural therapy, interpersonal therapy and thought field therapy), livelihood programmes, EMDR, art therapy, relaxation techniques, counselling	
Mechanism	Professional support Peer support Understanding emotional responses to trauma Helping others Reclaiming control	Safe spaces Mental health literacy Social connection Strong therapeutic relationship Gender-matching Cultural competency Empowerment
Outcome	Reduced PTSD, depression, anxiety, suicidal ideation and suicide attempts. Improved functioning scores, conduct behaviours, well-being	

The context is the setting, the mechanism is a change in response or reasoning of participants upon introduction of the intervention, and outcomes are intended or unintended consequences of the intervention (Trickey *et al*., 2018). Our initial programme theory took into account authors' current views on which mechanisms may lead to positive mental health outcomes. This theory was tested and refined against results from the literature and stakeholder interviews.

‘COVID-19’ was excluded from our search terms as initial literature searches were conducted prior to the pandemic outbreak. As such, realist semi-structured interviews with stakeholders who deliver psychosocial interventions to female Yazidi IDPs were used to explore the impact of COVID-19 on the population's mental health, and to test our CIMO. Data from these interviews were presented separately to explicitly acknowledge stakeholders' contributions to knowledge production, as recommended by a recent publication in the field of realist synthesis (Abrams *et al*., 2021).

Stakeholders' public email addresses were identified through internet searches of humanitarian institutions in the region who were likely to have information needed to support, refute or refine the programme theory (Wong *et al*., 2016). Thirty-three stakeholders were recruited from seventeen institutions and eight participated. The remainder either didn't respond or objected, stating that they had no time, or did not work within the institutions anymore. All were aged 18 or over, including men and women. No compensation was given for their time.

Due to their semi-structured nature, interviews lasted between 45 and 90 minutes. Interviews were conducted one-on-one by one of the authors in English. Five consented to being audio-recorded. Two provided answers in writing, due to language barriers, where responses were written with the help of an English-speaking colleague in their institution (*n* = 1) or using an online translator (*n* = 1). The remaining stakeholder preferred not to be audio-recorded, so notes were taken.

Informed consent was obtained from all participants. Explanations were provided on the research objectives and procedures; ample opportunities for clarification and right to withdraw participation were given. To ensure strict online safety, access to interviews was password protected. Interview data were stored in an encrypted document. Risks of distress associated with research participation were considered minimal as interviews focused on work experiences, and did not collect data of a more personal nature. Ethics approval for qualitative data collection was obtained from the UCL Research Ethics Committee [ref.: 18701.001].

Interview transcripts and notes were analysed for recurrent themes using thematic analysis (Braun and Clarke, 2006). This process was inductive: themes were coded without trying to fit them into a pre-existing framework. Identification of themes was semantic, based upon explicit views expressed.

Purposive sampling, involving iterative, snowballing techniques, was used to further develop, support, refute or refine our programme theory: what outcomes do psychosocial interventions delivered to female survivors of CRSV living in IDP settings have? What causes these outcomes (mechanisms)? (Wong *et al*., 2016). Records were also searched for interventions delivered to survivors of CRSV since the emergence of COVID-19, however no results were found. Sampling was complete at the point of theoretical saturation (Mogre *et al*., 2014). Records which met the eligibility criteria were included.

Literature was triangulated with interviews where similar themes were observed. One author analysed all data twice; where interpretation was difficult, the other author completed independent analysis, to reach a consensus. Table 2 outlines the refined programme theory.

## Results

**Fig. 1.**
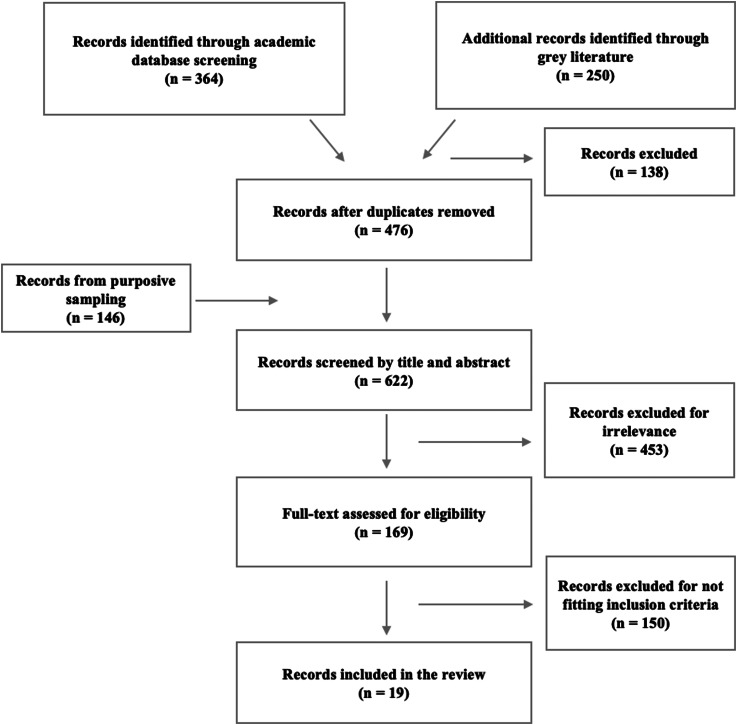
Search strategy and yield.

Figure 1 outlines the search strategy and yield. Nineteen records, spanning 2001–2020, were included. Interventions were delivered in nine post-conflict settings: KRI (*n* = 8), Uganda (*n* = 4), Nigeria (*n* = 1), Turkey (*n* = 1), Sri Lanka (*n* = 1), Bosnia (*n* = 1), Democratic Republic of the Congo (DRC) (*n* = 1), Rwanda (*n* = 1) and Burundi (*n* = 1).

Interventions consisted of art therapy (*n* = 2), eye movement desensitisation and reprocessing (EMDR) (*n* = 1), cognitive behavioural therapy (CBT) *v.* thought field therapy (*n* = 1), CBT (*n* = 1), interpersonal therapy (IPT) *v.* creative play (*n* = 1), narrative exposure therapy for children (KIDNET) *v.* meditation-relaxation (MED-RELAX) (*n* = 1), livelihood programmes (*n* = 2), trauma-focused CBT (TF-CBT) (*n* = 1), narrative exposure therapy (NET) (*n* = 1), IPT *v.* NET (*n* = 1), trauma workshop (*n* = 1), counselling (*n* = 2), multi-component intervention (*n* = 1), group therapy (*n* = 2) and a resilience programme (*n* = 1).

## Proposed underpinning mechanisms

We identified seven mechanisms which explain how interventions might work to improve the mental health and well-being of female survivors of CRSV in low-resource settings: safe spaces, a strong therapeutic relationship, empowerment, mental health literacy, social connection, gender-matching and cultural competency. Outcomes included reduced PTSD, depression, anxiety, suicidal ideation, attempted suicide and improved well-being. Table 3 outlines the results.

**Table 3.** Literature included in the review

Study	Study design, target population, sample size (*n* = ) and intervention	Mechanism							Outcomes
		Mental health literacy	Cultural competency	Strong therapeutic relationship	Safe spaces	Empowerment	Gender matching	Social connection	
(Abdulah and Abdulla, 2020)	Pre and post-test quasi-experimental study of art therapy delivered to Yazidis (females, aged 10–27) in Sharya camp, KRI (*n* = 14)								Decline in suicidal ideation score (16.71 → 6.50, p=0.002) Increased desire to live (0.86 → 1.93, p<0.0001) Passive/active suicide attempts stopped (p=0.012 and p=0.005)
(Acarturk *et al*., 2016)	Randomised control trial (RCT) of EMDR delivered to adults (males and females, aged ≥18) in Kilis Refugee Camp at the Turkish–Syrian border, Turkey (*n* = 70)*								Depression in intervention *v.* control group immediately post-treatment (10.45 *v.* 26.35, p<0.001) and at 1 month followup (12.85 *v.* 26.13, p<0.001) PTSD diagnosis in intervention *v.* control immediately post intervention: 39% *v.* 94%; 5 week follow-up: 51% *v.* 96%
(Atulomah *et al*., 2020)	Case-control study of motivational counselling delivered to IDPs (males and females, aged ≥60) living in 2 IDP camps, Borno State Nigeria (*n* = 40)								PTSD declined in intervention *v.* control group (3.50 → 2.60 *v.* 3.75 → 3.75 p<0.05). At 13 week follow-up: intervention group had higher PTSD symptom reduction than control group
(Bass *et al*., 2016)	RCT of trauma counselling and psychoeducation delivered to IDPs (males and females, aged ≥18) living in Dohuk governorate, KRI (*n* = 209)								Significant improvements in depression in intervention *v.* control group (pre-post change −0.83 *v.* −0.62, p=0.02), functional impairment (-0.76 *v.* -0.39, p=0.03) and anxiety (−0.64 *v.* -0.19, p=0.01)
(Bolton *et al*., 2007)	RCT of group IPT *v.* creative play delivered to adolescents (males and females, aged 14–17) in 2 IDP camps, northern Uganda (*n* = 304)								Significant decline in depression from baseline to follow-up in IPT intervention (42.9 → 24.9, p=0.002) *v.* control (44.2 → 36.1) but not in creative play Greater reduction in depression among girls than boys
(Catani *et al*., 2009)**	RCT of KIDNET *v.* MED-RELAX delivered to war-affected children (girls and boys, aged 8-14) in post-Tsunami IDP camps, Manadkadu, Sri Lanka (*n* = 31)								Significant reduction in PTSD diagnosis at 6 months follow-up (p<0.001): 81% of KIDNET group and 71% of MED-RELAX group did not reach threshold for PTSD diagnosis. No significant difference between groups
(Dybdahl, 2001)	RCT of semi-structured group therapeutic discussion delivered to mothers (aged 20-44) living in refugee settlements and private accommodation for IDPs in Tulza, Bosnia (*n* = 42)								Significant improvement in well-being (+1.93, *p*<0.05), sense of social support (+1.71, *p*<0.05), reduction in arousal (−2.00, *p*<0.05) in intervention *v.* controls
(Freij, 2018)	Qualitative evaluation (interviews and FGDs) of livelihood programme delivered to IDPs (males and females, aged 18–35) in Dohuk Governorate, KRI (*n* = 72)								42% of those surveyed stated that the intervention helped to improve their psychological well-being 98% said their relationships with families, friends and neighbours had improved
(Kaya and Luchtenberg, 2018)	Qualitative evaluation (interviews and FGDs) of livelihood programme delivered to IDPs (males and females, aged ≥18) in camps and settlements across KRI (*n* = 129)								Participants stated positive feelings of empowerment, independence and well-being through enrolment in the programme
(Lancaster and Gaede, 2020)	Pre and post-test experimental study of a resiliency programme delivered to IDPs (males and females, aged 12–98) in Khanke, Essien, Kabarto and Soran camps, KRI (*n* = 766)								Significant improvement in PTSD symptoms pre and post-intervention (29.41 → 13.32) and follow-up (14.42), p<0.001 Greater decline in female participants (35.81 → 12.85) than males (25.74 → 13.63)
(Nakimuli-Mpungu *et al*., 2013)	Quasi-experimental study of group therapy intervention delivered to war-affected IDPs (males and females, aged ≥18) in four trauma clinics, northern Uganda (*n* = 613)								Faster reduction in depression symptom scores in intervention *v.* control group at 6 months follow-up (−3.38 → −0.30), *p*=0.019] Faster reduction in PTSD scores in intervention group *v.* control group at 3 months (−4.21 → −0.10), *p*=0.042] 3-months follow-up: participants who attended ⩾2 sessions had faster increases in functioning scores than those who attended one session
(O'Callaghan *et al*., 2013)	RCT of TF-CBT delivered to war-affected girls (aged 12–17) rescued from brothels in Beni, DRC (*n* = 52)								TF-CBT group experienced reductions in PTSD from pre-post intervention (40.88 → 18.38, p<0.001) *v.* control group (40.29 → 42.93), and significant improvements in symptoms of depression/ anxiety (37.96 → 13.96, p<0.001) *v.* control (39.18 → 40.04)
(Schaal *et al*., 2009)	RCT of NET *v.* IPT delivered to youths (males and females, aged 14–28) living in child-headed households and orphanages in Kigali, Rwanda (*n* = 26)								6-month follow-up: 25% of NET and 71% of IPT participants fulfilled PTSD criteria Mean depression scores significantly decreased in both intervention types (IPT = 6.57 → 4.79, p=0.05; NET = 6.00 → 5.00, p=0.05)
(Schneider *et al*., 2018)	Case-control study of NET delivered to IDPs (males and females, aged 18-80) in former IDP camps (Anaka, Pabbo, Koch Goma) and villages (Gulu and Nwoya district), Uganda (*n* = 1131)								Reduction in prevalence of PTSD diagnosis following NET intervention for group with perceived stigmatisation at 4 months follow-up (166 → 60, p=0.015) and 10 months follow-up (166 → 15, p=0.004)
(Seidi *et al*., 2020)	Case series study of CBT *v.* thought field therapy (TFT) delivered to IDPs (males and females, aged 18-43) receiving treatment at two centres in Kalar City, KRI (*n* = 31)								Of 13 participants who received CBT, only one experienced improved PTSD and anxiety symptoms 11 participants who received TFT showed improvement in symptoms of PTSD and/or anxiety 7 participants who received CBT and showed no improvement then showed improvement when subsequently enrolled in TFT
(Sonderegger *et al*., 2011)	Case-control study of CBT delivered to IDPs (males and females, aged 15-56) living in Pabbo and Ajulu IDP camps, Uganda (*n* = 202)								Significant reduction in three depressive scales at post-assessment (*p*<0.001) and at 3-months follow-up (*p*<0.001) for participants in intervention group Significantly lower anxiety scores for intervention v control group (*p*<0.001), maintained at 3-months follow-up (*p*<0.001)
(The Lotus Flower, 2017)	Annual report assessment of art therapy delivered to IDPs (females, aged ≥18) in Rwanga camp, Dohuk governorate, KRI (*n* = 85)								Interviews with participants indicated improved well-being 85 women graduated from the intervention 35 earned an income through local contracts
(Womersley and Arikut-Treece, 2019)	RCT of psychotherapy, EMDR, art therapy, delivered to Free Yezidi Foundation Women's Centre members (females, aged ≥18) in Khanke IDP camp, Dohuk governorate, KRI (*n* = 200)								74% increase in self-reported WHO-5 well-being scale among service users PTSD decreased significantly upon completion of programme (81.25% → 45%)
(Yeomans *et al*., 2010)	RCT of 3-day trauma workshop with and without psychoeducation delivered to participants (males and females, mean age 38.6 years, SD =12.8) in two IDP camps in rural Burundi (*n* = 113)								Participants in workshop without psychoeducation had greatest reduction in PTSD (2.25 → 1.89) *v.* control (2.04 → 2.11), p<0.01 and *v.* those in workshop with psychoeducation (2.14 → 1.97), p<0.05
TOTAL		10	16	7	9	8	9	12	

Footnote: *Acaturk et al., 2016. Record was included despite intervention delivery to Syrian refugees as camp was located on the Syrian border. **Catani et al., 2009. Record was included due to exposure to war experiences, but note additional exposure to Tsunami.

## Mechanism 1: delivery of interventions in safe spaces

Delivery of EMDR at a camp-kindergarten, where participants could avoid stigma by pretending they were dropping off their children, provided a place for participants to engage with treatment without fear of rejection (Acarturk *et al*., 2016). Art-based interventions at a women's centre enabled female Yazidis to share experiences of sexual violence in a women's only environment (The Lotus Flower, 2017). Safe spaces were important for displaced women to attain livelihood skills, build social networks and express themselves (Kaya, 2018).

Focus group discussions (FGDs) with Yazidi females attributed delivery of interventions in a safe space as a key reason for their improved well-being (Womersley and Arikut-Treece, 2019). This was supported by one stakeholder: ‘*We work within a safe space and try to make it as accessible as possible…this could be a Community Centre or it could be a room in a youth centre*’ [Interview 3].

## Mechanism 2: strong therapeutic relationship

Beyond physical safety, survivors of CRSV need to feel emotionally safe to share their experiences. Staff who felt ‘like family’ were attributed to the success of a multi-component intervention with Yazidi females (Womersley and Arikut-Treece, 2019). In the DRC, participants attributed their improved PTSD, depression and anxiety scores to the delivery of TF-CBT by facilitators known to them (O'Callaghan *et al*., 2013). Trauma-based counselling, where emphasis was placed on therapeutic relationship, reduced depressive symptoms (Bass *et al*., 2016).

Two stakeholders necessitated a trusting therapeutic relationship for Yazidis due to their experiences of persecution: ‘*They went through 74 genocide alone, so they always have negative thoughts about their neighbours*’ [Interview 7].

## Mechanism 3: gender-matching

Due to the sensitive and gendered nature of CRSV, gender-matching of therapist and participant proved effective in reducing depression, PTSD and anxiety (Schaal *et al*., 2009; Sonderegger *et al*., 2011; Acarturk *et al*., 2016; Lancaster and Gaede, 2020). Delivery of IPT to small gender-homogenous groups, with gender-matched participants and therapists, significantly reduced depressive symptoms (Bolton *et al*., 2007).

The Yazidi community is male dominated [Interview 5], so gender norms tend to dictate engagement with psychosocial interventions: ‘*the man often refuses to engage in family therapy as men should be brave, should not cry and talk about himself*’ [Interview 4]. Societal stigma associated with CRSV means having a female therapist can allow women to share their experiences: ‘*For some of the Yazidis it makes a difference that you are a woman – if a male psychotherapist they wouldn't be able to talk about it due to the culture and gender norms*’ [Interview 4]; ‘*There are definitely people who prefer women… It's a lot easier for them to speak about their own personal details of such events with a female*’ [Interview 3].

However, an intervention delivered by a man resulted in long-lasting reductions in suicidal ideation and suicide attempts amongst Yazidi women (Abdulah and Abdulla, 2020). This could be explained by resource scarcity: ‘*Often we don't have female experts who have good training or information about treating patients therefore the survivor takes the male therapist*’ [Interview 8].

## Mechanism 4: cultural competency

Positive outcomes from gender-matching more broadly reflect the need for culturally competent intervention design and delivery. Interventions delivered by native facilitators reduced PTSD symptoms (Catani *et al*., 2009; Yeomans *et al*., 2010) and improved well-being (Dybdahl, 2001). Intervention delivery by community health workers, based on a curriculum developed by experts in Iraqi culture, reduced depression (Bass *et al*., 2016). Culturally-relevant activities increased participants' comfort (Sonderegger *et al*., 2011). A CBT intervention, when delivered to a largely illiterate population, showed minimal success when compared to thoughtfield therapy, which better suited participants' preference for traditional healing (Seidi *et al*., 2020). An art-based intervention allowed participants to understand their trauma in a way which made sense to them (Abdulah and Abdulla, 2020).

Delivering interventions in a culturally appropriate way helped to develop a trusting relationship: ‘*I also learnt Kurmanji (the main dialect used by Yazidis), so that I can speak to Yazidis and relate to them and build trust*’ [Interview 4].

However, a lack of trained facilitators is a major barrier: ‘*Very few people from the region who do understand cultural notions of health are trained. It's very rare to have someone who's able to adapt a model that is Western or that is foreign to the region*’ [Interview 3].

## Mechanism 5: social connection

Group interventions allowed participants to discuss their traumatic experiences, amounting in faster declines in depression and PTSD compared to non-treatment groups (Schaal *et al*., 2009; Nakimuli-Mpungu *et al*., 2013). Mothers with experience of severe war activities reported a positive sense of social support in group therapy (Dybdahl, 2001). Peer support was so central to an intervention's success amongst adolescent females that they formed small groups outside of the intervention, to practice techniques learned (O'Callaghan *et al*., 2013). Female Yazidis felt they had gained supportive relationships, improved self-esteem and psychological safety in an art-based intervention (Abdulah and Abdulla, 2020).

Stakeholders noted that group-based interventions ‘*allowed survivors to connect and learn from their peer groups…facilitating resiliency and emotional support from people who have experienced similar things, making them feel like they are not alone*’ [Interview 5]. This is particularly important for the Yazidi community due to their persistent experiences of violence which require ‘*collective healing because violence was collective in nature*’ [Interview 1]. Another stakeholder noted that there was a benefit to Yazidis engaging with others outside of their family: ‘*These group activities also activate an alternative social network rather than dwelling on their trauma within their families*’ [Interview 3].

However, stigma is a major challenge for group interventions: ‘*[stigma] is something that we deal with, on a day-to-day basis*’ [Interview 3]. Stigma associated with experiences of CRSV is particularly widespread: ‘*Society forced her to forget the past and what happened because it happened by people who are not of their religion. Forgetting what happened is believed to be better*’ [Interview 1].

Individual psychotherapy was therefore deemed more beneficial [Interview 3 and 8], because ‘*they feel more comfortable if they don't have to talk about people they know or family members*’ [Interview 8]. Art therapy also helped to heal from trauma without having to speak about it: ‘*Survivors can express themselves better and share what they cannot speak about in the community in their drawing*’ [Interview 1].

## Mechanism 6: mental health literacy

Integrating services with psychoeducation was another means to engage Yazidis and overcome stigma [Interview 7]. Mental health literacy, achieved through psychoeducation, reduced PTSD symptoms (Schneider *et al*., 2018). Psychoeducation delivered to guardians of sexually-exploited orphans helped to re-establish contact with family and reduce stigma (O'Callaghan *et al*., 2013; Acarturk *et al*., 2016).

However, where psychoeducation was replaced by a workshop on safety and trust, participants experienced greater reductions in PTSD. Reduced effectiveness of psychoeducation could be due to participants' increased engagement with their trauma, and so greater risk of re-traumatisation (Yeomans *et al*., 2010).

Stakeholders supported the need for mental health literacy: ‘*Yazidi women are illiterate as women aren't allowed to go to school and don't know what psychological disorders are…They just hear that they are crazy and that you shouldn't go to a psychiatrist because people will know that you are crazy. This means you need to do psychoeducation for these terms*’ [Interview 5].

## Mechanism 7: empowerment

Art-based interventions reduced suicidal ideation by empowering participants to feel like they had a meaningful purpose: ‘I was so happy in the course, because I was thinking of being an artist’ (Abdulah and Abdulla, 2020); ‘I now have my own sewing machine…my hope for the future is to become a highly skilled tailor who can afford a good life for my children’ (The Lotus Flower, 2017). An intervention which concluded with a graduation ceremony, where participants' guardians watched them receive certificates, led to significant reductions in depression and anxiety (O'Callaghan *et al*., 2013).

Livelihood interventions reduced depressive symptoms and allowed Yazidi women to ‘*get a job in the future and build selfreliance… and increase life skills*’ [Interview 1] (Bass *et al*., 2016; Kaya and Luchtenberg, 2018). This is particularly important because ‘*ISIS's attack caused the survivors to deny their wishes and ability to have a future*’ [Interview 1]. This narrative has extended beyond the attack: Western media has ‘*focused on Yazidis (and especially women) as weak victims. Many staff therefore find it challenging to help them move past the idea that they are victims*’ [Interview 3]. The ability to gain skills has also relieved some of the ‘double burden’ of stress related to ‘*poverty after the loss of everything from the ISIS attack*’ [Interview 1].

### The impact of COVID-19 on mechanisms

Table 4 outlines the findings from stakeholder interviews on the impact of COVID-19 on Yazidi females. Interviews revealed that Yazidi females have experienced increased flashbacks, PTSD, depression and anxiety since the start of the pandemic. We suggest that a change in the context and delivery of interventions has subsequently affected mechanisms and outcomes within our CIMO configuration. Closure of safe spaces, online delivery of interventions, isolation from social networks, redistribution of support workforce, loss of livelihoods, and increased rates of IPV have contributed to Yazidis' suffering.

**Table 4.** The impact of COVID-19 on our CIMO configuration

Component	Refined programme theory	After onset of COVID-19
Context	Conflict-affected females who are internally displaced in low-resource settings. Have experience of historic and collective trauma, social and gendered inequality, live in poor displacement conditions with poor access to services	Conflict-affected females who are internally displaced in low-resource settings. Have experience of historic and collective trauma, social and gendered inequality, live in poor displacement conditions with poor access to services, during a global pandemic
Intervention	Group and individual psychotherapy (narrative exposure therapy, cognitive behavioural therapy, interpersonal therapy and thought field therapy), livelihood programmes, EMDR, art therapy, relaxation techniques, counselling	
Mechanism	Safe spaces Mental health literacy Social connection Strong therapeutic relationship Gender-matching Cultural competency Empowerment	**Therapeutic relationship:** movement restrictions have resulted in road closures for many months [Interview 6] and an inability of service providers to deliver in-person care: ‘*Regarding going to the field or inside the community centres, no one was allowed to do that*’ [Interview 7]. Organisations have tried to keep camp members connected to services using portable devices, however ‘*some NGOs stopped totally without conducting any online services*’ [Interview 7]. Unstable internet connections and frequent electrical shortages mean that many have limited access to regular support [Interview 3]. Restrictions also meant it was ‘*difficult communicating with the work team because all of our meetings and communication went online*’ [Interview 6]. **Safe spaces:** If able to engage in online support, many feared speaking about their experiences at home [Interview 6]. Increased time at home due to lockdowns also increased rates of IPV: ‘*Patients are more isolated which increases stress and rates of violence in the family. We have seen increases in domestic violence and violence against children…the issue with mobile delivery is that people have no privacy – if you are working with a Yazidi woman in a tent, she has suffered ISIS abuse but it is also likely that she has suffered abuse in her household. Some people we've been working with have protested and said they can't work over the phone anymore*’ [Interview 5]. To deal with the closure of safe spaces, one stakeholder noted that they had ‘*converted some centres used for art activities into consultation rooms where clients can come in and speak to staff remotely*’ [Interview 3]. Another has been working on ‘*strategic planning which involves moving video conferencing to centres rather than leaving them in camps*’ [Interview 5]. **Social connection:** despite evidence of an increased prevalence in common mood disorders since the outbreak of the pandemic, group-based interventions have been reduced from 20 participants to 4 or 6 [Interview 2]. ‘*This is necessary to prevent spread of the virus because in the camp, the tents are close to each other*’ [Interview 1]. Sewing and drawing classes were reduced to 5–6 participants [Interview 1] **Mental health literacy:** interventions have mostly focused on mental health disorders surrounding COVID-19 and neglected the stigma associated with other forms of trauma, such as CRSV: ‘*The only activity that we were having was COVID-19 psychoeducation*’ [Interview 7]. **Gender-matching:** redirected human resources to COVID-19 mean gender-matching has not been prioritised. Stakeholders did not provide specifics but spoke generally about reallocation of resources due to the pandemic. **Empowerment:** loss of livelihoods and economic strain meant ‘*higher levels of deprivation and stress –almost no one has access to work*’ (Interview 5); ‘*[they fear] they might not be able to work and get money for the family if they get COVID-19*’ [Interview 7]. ‘*The curfew also meant they might not be able to go to Dohuk for work*’ (Interview 8). Being unable to protect loved ones from the virus has also reactivated feelings of helplessness experienced during genocidal attacks [Interview 7]. **Cultural competency:** urgent public health messaging failed to integrate cultural factors such as high illiteracy rates: ‘*Some were saying it [COVID-19] is a political and economic issue that has been created to get money and such things. But then, day-by-day, the cases were increasing. So then they realised that it's true. But there are also factors which impact on that, for example, an educated person will not be like an illiterate one. IDPs they are not educated. Yeah, they are illiterate*’ [Interview 7].
Outcome	Reduced PTSD, depression, anxiety, suicidal ideation and suicide attempts. Improved functioning scores, conduct behaviours, well-being	**Regressed treatment progress:** ‘*It has affected treatment and therapy sessions. Many who were getting better, COVID-19 came and damaged all the progress.. Some are afraid of COVID-19 but most would rather receive mental health treatment – they want someone to help*’ [Interview 8]. **Symptom relapse**: ‘*Cases who suffered from PTSD and flashbacks, the interventions made them stable but COVID-19 means they had to stay in tents for 3 months and this caused their mental health to decline. Their homes aren't like houses but are poor living conditions. When they don't go out for a few weeks, their flashbacks returned*’ [Interview 8]. **Fear of attack:** ‘*There have been 20 attacks by Turkey in towns and near camps. Many ISIS fighters were Turkish in origin, so when we heard that Turkey was coming, we know ISIS will come with them*’ [Interview 7]; ‘*Some of the women were afraid because of the memories of genocide*’ [Interview 8]. **Increased symptoms** including flashbacks, boredom, frustration, shortness of breath, loss of energy, nightmares, fear, suicidal thoughts, PTSD, depression, anxiety [Interviews 1, 3, 7 and 8]; ‘*COVID-19 has affected cases very badly*’ [Interview 8]; *The women understand what is happening and they are really scared*” [Interview 5]; ‘*Since the start of the Coronavirus, suicide for young men and girls have been detected*’ [Interview 8]. **Increased desire to emigrate abroad** [Interview 1] **or anxiety about whether to return to Sinjar**: ‘*Some others were saying that if they didn't come back to our home town [Sinjar], they will not be able to go back again. So, they were having a big anxiety about that*’ [Interview 8]. **Lack of trust in authorities**: ‘*Some people don't believe that COVID-19 is real, it is political*’ [Interview 8]; ‘*There are allegations and rumours that more and more cases are in camps and authorities are not reporting them*’ [Interview 3].

## Discussion

We aimed to address the evidence gap on how interventions work to improve the mental health of displaced female Yazidi survivors of CRSV in the KRI. A realist review of psychosocial interventions delivered to female survivors of CRSV identified seven mechanisms: safe spaces, a strong therapeutic relationship, empowerment, mental health literacy, social connection, gender-matching and cultural competency. Interviews with stakeholders who deliver psychosocial interventions to female Yazidis in the KRI confirmed relevance of these mechanisms. Interviews also confirmed that COVID-19 has worsened Yazidi survivors' mental health.

### Mechanisms in the research context

Safe spaces should be an essential component of interventions delivered to female Yazidi IDPs, whose experiences of persistent marginalisation continue to threaten their existence (Persecution Prevention Project, 2019). Displacement means they are unable to express their Yazidi identity, nor are they safe to return to home. The ongoing captivity of 3500 women and 1200 children by ISIS is a stark reminder of continual genocidal ideology in the region, and the inability of Iraqi authorities to protect the community (Persecution Prevention Project, 2019; Womersley and Arikut-Treece, 2019). ISIS's destruction of Yazidi shrines means there are no longer places to practice their rituals and customs (Isakhan and Shahab, 2020).

Aside from violence perpetrated by other groups, Yazidi females are exposed to violence in their interpersonal relationships. In 2019, 66% of female Yazidi IDPs reported past-year exposure to IPV (Goessmann *et al*., 2020). These high rates are situated within the broader context – Iraq has the largest social and legal gender inequalities in the world (The World Bank Group, 2019). For a state which has so frequently been fractured by war, a ‘culture of violence’ has become normalised and women's bodies are readily exploited (Heise, 1998; Ahram, 2019).

It is therefore unsurprising that the closure of safe spaces due to COVID-19 has resulted in alarming increases in IPV. In one study, 346 married women in the KRI reported an increase in IPV from 32.1 to 38.7% (Mahmood *et al*., 2021). Stakeholder interviews echoed this pattern of increased violence within the Yazid community.

However, nurturing feelings of safety extend beyond the physical space. *Feeling* ‘safe to tell’, a mechanism previously reported by Spangaro (2015), is particularly important for Yazidi survivors of CRSV, where sexual relations with those outside of the community are condemned. Fear of discrimination has been attributed to suicide and mental illness (Goodman *et al*., 2020). One study illustrated that 44.6% of formerly enslaved Yazidi females felt extremely excluded by community members, and 32.3% felt worried about not being able to get married or continue their marriage (Erdener, 2017).

As such, a strong therapeutic relationship is a suitable mechanism to help Yazidi survivors to speak of their trauma and has been attributed to positive intervention outcomes in non-conflict settings (Cloitre *et al*., 2002; Keller *et al*., 2010). However, applicability of this mechanism needs testing, for persistent persecution has amounted in a strong distrust of others, limiting engagement with psychosocial support services (Strang *et al*., 2020). As expressed by one care provider: ‘Some of my patients do not believe we can help them at all. Earning their trust is the most difficult challenge’ (Jiyan Foundation for Human Rights, 2017).

Gender-matching could be one effective mechanism to support the building of trust, particularly for survivors of gender-based violence, where the abuse itself is centred around gendered identity (Ward and Marsh, 2006). A gendered preference for health-seeking has already been established amongst Yazidis, attributed to high prevalences of IPV and gynaecological issues (Cetorelli *et al*., 2017*a*).

Other important cultural factors must also be taken into consideration to aid the safe and effective delivery of interventions. Western models of mental illness are incompatible with the way Yazidis view suffering: distress is often described as physical in origin, such as ‘liver burning’ (Womersley and Arikut-Treece, 2019).

However, to scale up culturally competent interventions requires considerable resources. As home to one-third of Iraq's oil resources, the KRI is a region of ongoing conflict and limited responsibility is assumed for minority populations (The Kurdish Project). Economic under-development, emigration of healthcare professionals, poor management and war damage are widespread (Al-Khalisi, 2013).

To approach this issue, group interventions appear to be one cost-effective way of dealing with large numbers of individuals who need support (Nakimuli-Mpungu *et al*., 2013; van Westrhenen *et al*., 2017). Social connection is one of the most consistent predictors of psychological adaptation following a range of traumatic events, including forced displacement (Shishehgar *et al*., 2017). Social connection is culturally relevant for Yazidis, whose collective mourning is a common coping behaviour for their collective trauma, and reflects the behaviour of other genocide survivors (Kanyangara *et al*., 2007; Erdener, 2017; Arikut-Treece, 2019).

Where stigma is a common challenge to group interventions, mental health literacy can help to normalise survivors' feelings and improve access to psychological services (Bosqui and Marshoud, 2018). A lack of knowledge about mental health problems is one of the key barriers to help-seeking amongst children and adolescents in high-income countries (Radez *et al*., 2021). For Yazidis, illiteracy has been directly associated with suicide and mental illness amongst women and girls, attributed to their inability to source and access services (International Organization for Migration, 2011).

Greater engagement with services can also empower individuals to seek employment, resulting in higher incomes and a positive effect on mental health (Thomson *et al*., 2022). This effect is especially profound in displacement settings; a loss of jobs since the outbreak of COVID-19 has been reported as a main cause of suicide in the Yazidi community (van Wilgenburg, 2021).

However, true empowerment of survivors begins with acknowledgement of their own perceptions and priorities, which may be different from what is expected by the outside world (Akhavan *et al*., 2020). Responding to the mental health needs of female Yazidis requires a greater understanding of their day-to-day experiences.

### Strengths and limitations

The findings of this review are from a multitude of studies, in a diverse range of low-resource settings. Utilisation of grey literature and peer-reviewed literature resulted in a large pool of data within which to build the CIMO configuration.

However, records included interventions delivered to both males and females, thus limiting the applicability of findings solely to female Yazidis. The purposive sampling strategy does not require an exhaustive search of databases, exposing the potential to partial or incomplete results (Pawson *et al*., 2005; Kiss *et al*., 2020). Only a small number of Western mental health terms were used in searches. Despite PTSD, according to DSM-V criteria, being identified as a valid measure amongst IDPs living in Iraqi Kurdistan (Ibrahim *et al*., 2018), the dominant use of DSM-PTSD diagnoses as an entry criteria in review contexts where it has not been approved excludes those who do not meet Western standards of mental illness (Patel *et al*., 2014). The search may have missed records with titles and abstracts in languages other than English (Spangaro *et al*., 2013).

Interviews were based upon one-time consultations with a small sample. Questions may therefore elicit a different response at a later date. Although the study was designed to explicitly recognise the perspectives and contributions of service providers, we did not provide an opportunity for interviewees to offer their feedback on the interpretation of findings.

## Conclusion

We explored how global evidence on psychosocial interventions delivered to female survivors of CRSV applies to the internally displaced female Yazidi population. Seven mechanisms underpin psychosocial interventions delivered to displaced female survivors of CRSV: safe spaces, a strong therapeutic relationship, social connection, mental health literacy, cultural competency, gender-matching and empowerment.

Realist semi-structured interviews confirmed relevance of mechanisms to female Yazidi IDPs in the KRI. Interviews highlighted the impact of COVID-19 on the mental health of this population. Increased flashbacks, regressed treatment progress and increased fear were documented. Closure of safe spaces, isolation from social networks, redistribution of resources, disruptions to in-person interventions, loss of livelihoods and increased rates of IPV have contributed to Yazidis' suffering.

COVID-19 is just one challenge affecting the implementation and delivery of interventions. Gendered and legal inequalities, fractured governance and war damage are just some. Future research should focus on understanding the daily experiences of female Yazidi IDPs, and would benefit from large-sample quantitative analysis of mental health scores pre- and post-pandemic. Research should also investigate which interactions of mechanisms are most effective for this population.

## Data Availability

Not applicable to support anonymisation of interview data.

## Supplementary material

For supplementary material accompanying this paper visit https://doi.org/10.1017/gmh.2022.55. click here to view supplementary material
