# Supplementary material for: The mental health and well-being of internally displaced female Yazidis in the Kurdistan Region of Iraq: a realist review of psychosocial interventions and the impact of COVID-19
Source: Glob Ment Health (Camb). 2022 Nov 4;9:508–20. doi: 10.1017/gmh.2022.55 (PMC9806966; doi:10.1017/gmh.2022.55)
Supplement: Supplementary file 1 [file S2054425122000553sup001.docx]

**Supplement to:**

**The mental health and well-being of internally displaced female Yazidis in the Kurdistan Region of Iraq: a realist review of psychosocial interventions and the impact of COVID-19.**

**Corresponding author:**

Sophia Lobanov-Rostovsky

Institute for Global Health

University College London

WC1N 1EH, UK

Email: [sophia.lobanov-rostovsky.14@ucl.ac.uk](mailto:sophia.lobanov-rostovsky.14@ucl.ac.uk)

**APPENDIX**

Contents

[Supplementary figure 1: Mental health needs of internally displaced, female Yazidis in the Kurdistan Region of Iraq 3](#_Toc104555927)

[Supplementary table 1: Summary of literature review search strategy and selection, according to RAMESES standards of reporting for realist reviews 4](#_Toc104555928)

[References 5](#_Toc104555929)

**Supplementary figure 1: Mental health needs of internally displaced, female Yazidis in the Kurdistan Region of Iraq. Adapted from (Miller and Rasmussen, 2017).**

**
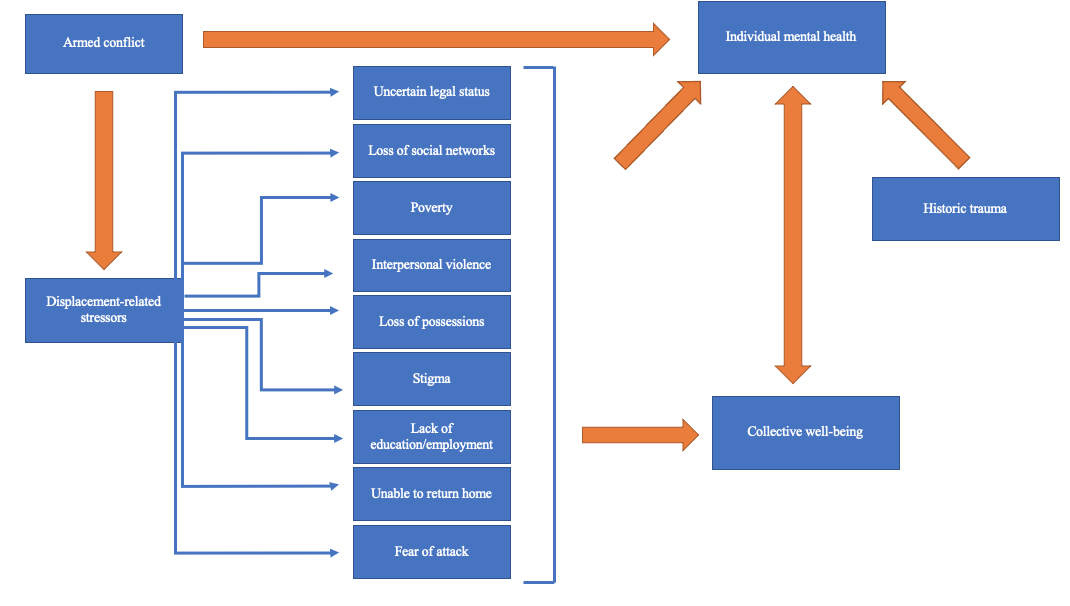
**

# **Supplementary table 1: Summary of literature review search strategy and selection, according to RAMESES standards of reporting for realist reviews (Wong et al., 2013).**

| Section and topic | Description |
| --- | --- |
| Title | Our manuscript title clearly outlines that it is a realist review: *The mental health and well-being of internally displaced female Yazidis in the Kurdistan Region of Iraq: a realist review of psychosocial interventions and the impact of COVID-19.* |
| Abstract | The abstract provides a brief description of: the study’s background, research objectives, search strategy, methods and results. Our conclusion outlines the implications of this research for intervention implementation and delivery. |
| Introduction | Our introduction begins by summarising current evidence on the topic area, focusing on the magnitude and risks of conflict-related sexual violence and its mental health implications in a global context. We then detail the research context: internally displaced, female Yazidis in the Kurdistan Region of Iraq in a COVID-19 context. We explicitly outline our objectives of the paper at the end of the introduction. |
| Methods | The methods section of this paper describes the function of a realist review and how it is the most suitable method to use for our research objectives. We explain the process used in exploratory scoping, including which databases were screened and which terms were used. We justify using an exploratory scoping of the literature in order to formulate our context-intervention-mechanism-outcome (CIMO) configuration. Table 1 provides the search terms and eligibility criteria used for scoping of the literature. The footnote to Table 1 outlines the grey literature sources used. A description is provided of how stakeholders who deliver interventions to the target population were identified and recruited for realist interviews. A thematic analysis was undertaken of notes and interview transcripts to identify recurrent themes for inclusion and refining the CIMO configuration, detailed in Table 2. At the point of theoretical saturation, purposive sampling through iterative searches and snowballing techniques was stopped. After a review of titles and abstracts, we extracted data through a process of note-taking and conceptualisation. Records which met the inclusion criteria were included in the final review. |
| Results | Figure 1 outlines the search strategy and yield of this review, including the number of records assessed for eligibility and included in the final review, reasons for exclusion and source of origin (academic database searches, grey literature, purposive sampling). Key findings are represented in Table 3 where each study is summarised by its study design, target population, sample size and intervention, the mechanisms identified for the study intervention(s) and outcomes. A description of results is written at the beginning of the results section including the number and type of different post-conflict settings, interventions, mechanisms identified and outcomes. We also outlined results from interviews about the mental health of Yazidis since the onset of COVID-19. |
| Discussion | We provide a general interpretation of our results and discuss implications of these results for the context of the internally displaced, female Yazidi population in the Kurdistan Region of Iraq. We provide ongoing reference of results to the review’s objectives. Strengths and limitations are discussed at the end of the discussion and explains where further work is needed. |
| Conclusion | Our conclusion lists the main findings and implications within the research context and recommendations for future research. The declarations section outlines the ethics approval and consent to participate, consent for publication, availability of data and materials, conflicts of interest, funding source and authors contributions. |

**References**

**Miller, K & Rasmussen, A** (2017). The mental health of civilians displaced by armed conflict: an ecological model of refugee distress. *Epidemiology and psychiatric sciences*, 26, 129-138.

**Wong, G, Greenhalgh, T, Westhorp, G, Buckingham, J & Pawson, R** (2013). RAMESES publication standards: realist syntheses. *BMC medicine*, 11, 1-14.
